# Development of a depot formulation with an *in situ* non-lamellar liquid crystal-forming system with phospholipids

**DOI:** 10.3389/fddev.2023.1270584

**Published:** 2023-10-19

**Authors:** Hiroaki Todo, Rina Niki, Akie Okada, Ibuki Narita, Kazuya Inamura, Ayu Ito, Shoko Itakura, Ichiro Hijikuro, Kenji Sugibayashi

**Affiliations:** ^1^ Faculty of Pharmacy and Pharmaceutical Sciences, Josai University, Sakado, Japan; ^2^ Farnex Incorporated, Yokohama Joint Research Center, Yokohama, Japan; ^3^ Faculty of Pharmaceutical Sciences, Josai International University, Togane, Japan

**Keywords:** lipid-based depot formulation, *in situ* forming system, sustained release, non-lamellar liquid crystal, long-acting drug delivery system

## Abstract

Non-lamellar liquid crystal (NLLC) structures have gained increasing attention for the controlled release of entrapped drugs. In the present study, an *in situ* NLLC structure-forming depot formulation through contact with water was developed using a ternary mixture system of soya phosphatidyl choline (SPC), 1, 2-dioleoyl-*sn*-glycero-3-phosphoglycerol sodium salt (DOPG), and sorbitan trioleate (Span 85), and the long-term release of an entrapped model drug, leuprolide acetate (LA), was investigated using evaluation of *in vitro* release and *in vivo* blood concentration–time profiles. Polarized images and small angle X-ray scattering analysis were used to confirm the presence of NLLC structures by contacting the prepared formulation with water. In addition, LA release and blood concentration–time profiles were investigated using *in vitro* and *in vivo* experiments, respectively. *In situ* NLLC constructed formulations by contacting water were achieved using a ternary mixture of SPC, DOPG, and Span 85. In particular, negative curvature was increased with an increase in the amount of Span 85 in the formulation, and an *Fd3m* structure was obtained with a sustained release of LA. A maintained blood concentration of LA over 21 days was confirmed by subcutaneous (*s.c.*) administration of the formulation. No retained administered formulation at the injection site was confirmed 28 days after administration without any signs of irritation, inflammation, or other apparent toxicity confirmed by visual observation. This result may be helpful for the development of a lipid-based formulation of peptides and proteins with sustained drug release.

## Introduction

Self-injectable formulation that enables extend-drug release such as depot formulation has been increasing attention because it can improve patients’ quality of life and reduces the risk of recurrence or deterioration of symptoms. In addition, it would be helpful to reduce the burden on healthcare professionals (Lim et al., 2022). Polymer-based depot formulations such as poly (lactic-co-glycolic acid), PLGA, has been already used, and various PLGA-based long-acting drug products have been approved by the U.S. Food and Drug Administration (FDA) for therapeutic delivery. However, PLGA-based formulations are difficult due to their complex manufacturing process, and most of these formulations exhibit initial burst release followed by slow and incomplete release (Lim et al., 2022).

Lipid formulations, such as liposomes and lipid nanoparticles, have gained increasing attention as vehicles for controlled drug delivery (Seo et al., 2023). Recently, non-lamellar liquid crystal (NLLC) technology has emerged as a novel formulation with sustained release properties (van ’t Hag et al., 2017). NLLCs composed of amphiphilic lipids can be classified into discontinuous cubic phases, such as primitive (*Im3m*), diamond (*Pn3m*), and gyroid phases (*Ia3d*), inversed hexagonal phase (*H2*), and discontinuous micellar cubic phase (*Fd3m*) based on their assembly shape (Muszynski et al., 2018). The critical packing parameter (CPP) is used to explain the shape of the amphiphiles, which determines the type of their construction form. The CPP is calculated by *V*/*al*, where *V* is the hydrophobic chain volume, *a* is the cross-sectional area of the hydrophilic head group, and *l* is the cross-sectional area of the hydrophobic chain length in the molecule. The drug release rate of the loaded drug is affected by the constructed structure of the NLLC. In particular, *Fd3m*, which has a closely packed inverse micelles structure, displays slower drug release compared with other NLLC structures. *In situ* systems that form NLLC structures by contacting water in body fluids may be a preferable formulation for drug delivery with microneedles and needle-free injectors.

Amphiphilic lipids form an NLLC structure, with glycerol monooleate and glycerol dioleate, mono- and di-oleic ester compounds with glycerol as a hydrophilic group having been used (Nielsen et al., 1998). Because these NLLC forming lipids have CPP >1, higher negative curvature of the membrane is induced by the addition, and many strategies have been reported for engineering structures that are a compositional mix of various lipids with different CPPs (Martiel et al., 2014). Changes in the constructed structure and diameter of aqueous channels in the structure are methods to control drug release.

In our previous study, a novel NLLC-forming lipid, mono-O-(5, 9, 13-trimethyl-4-tetradecenyl) glycerol ester, MGE, was used on contact with body fluids (Okada et al., 2021). The formulation consisting of MGE, 1, 2-dioleoyl-sn-glycero-3-phosphoglycerol sodium salt, DOPG, showed that the blood concentration of LA was maintained for 21 days or more after administration. However, a higher injection force was required for injection. As the injection force is a very important parameter for self-injectable formulations (Okada et al., 2021), the injection force should be considered when developing a self-injectable formulation.

Recently, non-ionic surfactants of sorbitan fatty acid esters, such as sorbitan monooleate, have been used as alternative lipids to fabricate NLLC structures (Baez-Santos et al., 2016). Sorbian fatty acid esters are cheap nonionic surfactants, commonly used as food emulsifiers, in cosmetic and pharmaceutical products. In particular, sorbitan trioleate (Span 85) has a large lipophilic moiety and is used as an excellent emulsifier. Liposomes composed of a phospholipid bilayer (CPP = 1) are traditionally used as drug delivery systems, and recently liposomal formulations have been utilized in a depot-injectable formulations (Ki et al., 2014; Rahnfeld and Luciani, 2020).

Controlled drug release by adding Span 85 to phospholipid-based formulations to form NLLCs by inducing higher negative curvature may be an effective procedure. Therefore, in the present study, phospholipid-based gel formulations that turn into an NLLC structures by contacting water in body fluids, including soya phosphatidyl choline (SPC), 1,2-dioleoyl-sn-glycero-3-phosphoglycerol sodium salt (DOPG), and Span 85 were prepared, and leuprolide acetate (LA) was entrapped as a model drug with a middle-sized molecular weight. In addition, injectability force was also investigated to show the usefulness of a self-injectable formulation.

## 2 Materials and methods

### 2.1 Materials

LA was purchased from Shin Nippon Yakugyo K.K. (Tokyo, Japan). SPC was purchased from Funakoshi Co., Ltd. (Tokyo, Japan). Span 85 was purchased from Kanto Chemical Co., Ltd. (Tokyo, Japan). DOPG was purchased from NOF Corporation (Tokyo, Japan). Other reagents and solvents were of special grade, and further purification was not conducted.

### 2.2 Animal experiments

Male Wistar rats (body weight 200 ± 20 g, 8 weeks old) were purchased from Sankyo Lab Service Co. Rats were kept in a room regulated at 25°C ± 2°C with a 12-h light/dark cycle (on, off time: 9:00–21:00). The rats were also allowed to consume water and feed *ad libitum*. Feed was purchased from MF Oriental Yeast Industry Co. Experimental animals were handled in accordance with the Josai University Experimental Animal Regulations after obtaining approval from the Josai University Ethics Committee (JU22005).

### 2.3 Preparation of formulations

Different proportions of SPC/DOPG/Span 85 were prepared at room temperature. The percentage compositions of the prepared formulations are shown in Table 1. The formulations were prepared by direct mixing of SPC/DOPG/Span 85. SPC was previously dissolved in 50 µL of ethanol The preparation procedure was as follows: SPC was completely dissolved in ethanol, then SPC/ethanol solution, Span 85, DOPG were weighed (ex. 50 μL of ethanol was added to 85 mg of SCP to obtain SPC/ethanol solution, and then 5 mg of DOPG and 10 mg of Span 85 were mixed with SPC/ethanol solution). LA was added at 3.75 mg per 100 µL of the obtaining lipid solution, and the mixed formulation was stirred for 1 h in a vial at room temperature. The obtained lipid solution containing LA was used for all evaluations in the present study. The phase behavior was confirmed using a polarized microscope.

**TABLE 1:** Formulations and their compositions in these experiments.

Formulation code	SPC	DOPG	Span 85
F_85:5:10_	85	5	10
F_70:5:25_	70	5	25
F_55:5:40_	55	5	40
F_75:15:10_	75	15	10
F_60:15:25_	60	15	25
F_45:15:40_	45	15	40
F_65:25:10_	65	25	10
F_50:25:25_	50	25	25
F_35:25:40_	35	25	40
F_25:25:50_	25	25	50
F_12.5:25:62.5_	12.5	25	62.5
F_55:35:10_	55	35	10
F_25:50:25_	25	50	25
F_12.5:75:12.5_	12.5	75	12.5

### 2.4 Polarized microscope observation

A microscope (VHX-5000, Keyence Corp., Osaka, Japan) was used for observation of polarized images of the prepared formulation. The observation was performed 5 min after applying 10 µL of each prepared formulation onto a slide glass with a high-viscosity dispenser (M10, M&S Instruments Inc., Osaka, Japan), followed by dropping an equal volume of phosphate-buffered saline (PBS, pH 7.4) onto the formulation. Immediately after applying PBS, the samples were covered with a cover glass and then observed at room temperature.

### 2.5 Small angle X-ray scattering (SAXS) analysis

A small angle X-ray diffraction instrument (Nano-Viewer, Rigaku Co., Ltd., Akishima, Tokyo, Japan) was used to confirm the structure. Measurements were performed at 30 kV and 40 mA (CuK α radiation, *λ* = 1.5418 Å). The focal length of the camera was set to 700 mm. Preformulations were immersed in 3 mL of PBS for 4 h, and measurements were performed at Kanazawa University (Kanazawa, Ishikawa, Japan). The obtained pattern was analyzed as follows (Okada et al., 2020). Briefly, the obtained X-ray pattern was analyzed by a Rigaku NANO-Solver program. All operation was conducted by a qualified researcher at Kanazawa University (Kanazawa, Ishikawa, Japan). The crystalline interplanar spacing parameter, *d*, was determined using the Bragg equation.

### 2.6 *In vitro* LA release from the formulations

LA release experiments from the prepared formulations were conducted using the dialysis method. Each formulation (100 μL) was placed into dialysis tubing (Pur-A-LyzerTM Mini 12,000 dialysis kit 25, molecular cut-off 12,000, Sigma Aldrich, St. Louis, MO, United States). Each formulation (100 μL) was placed in a dialysis tube (Pur-A-LyzerTM Mini 12,000 dialysis kit 25, molecular cut-off 12,000, Sigma Aldrich, St. Louis, MO, United States). The dialysis tube loaded with fromulation was immersed in 20 mL of PBS in a centrifuge tube (25 mL, centrifuge tube Mini MINI-2362-025, AGC Technoglass Co., Ltd., Shizuoka, Japan). The release experiment was conducted over 7 days, and during the experiment the centrifuge tube was set in a water bath at 37°C ± 0.02°C. The solution outside the dialysis membrane, receiver solution, was stirred with a stir bar during the experiment. Periodically, 500 µL of PBS was sampled from the solution outside the dialysis membrane, receiver compartment, and the same amount of fresh PBS was added to the receiver compartment to maintain a constant volume. Before each sampling, the receiver solution was agitated using a pipet for 10 s. The cumulative percentage of LA released was calculated by the loaded LA in the formulation.

### 2.7 Evaluation of injectability force of the prepared formulation

Injectability force of the prepared formulation was tested by a texture analyzer equipped with a 10 kg load cell (EZ Test, Shimadzu Corporation, Kyoto, Japan). Compression model was used for the analysis with a 1 mL syringes connected with a 23G needle. The formulation was filled into the syringe and the test was conducted at a speed of 1 mm/s. The maximum force (N) was determined when loaded force showed a constant value over the time. All measurement were carried out in triplicate.

### 2.8 *In vivo* experiments

Rats were cannulated in the jugular vein 1 day before the experiment. The prepared formulation was injected subcutaneously through a 23G needle in the dorsal region. The injection site was shaved prior to injection. Blood samples (100 μL) were taken periodically through the cannula until 28 days after injection. The obtained blood samples were centrifuged (21,500 × g, 5 min, 4°C) to obtain plasma. Plasma samples were stored at −80°C until assayed. LA solution was dissolved in saline containing 5% dimethyl sulfoxide at a concentration of 37.5 mg/mL, and the prepared formulation was *s.c.* injected at a dose of 18.75 mg/kg. The area under the curve over 21 days after administration (*AUC*_21days_) was calculated using the trapezoidal method.

### 2.9 LA determination

The samples obtained from the *in vitro* release experiment were mixed with acetonitrile at a ratio of 1:1 (v/v) by vortexing for 5 min, and the resulting solution was used as the measured sample. Plasma samples obtained in the *in vivo* experiment were mixed with acetonitrile at a 1:1 (v/v) ratio by vortexing for 5 min and then centrifuged (21,500 × g, 5 min, 4°C). The upper layer of the resulting solution was used as the measurement sample.

A liquid chromatography–tandem mass spectrometry (LC-MS/MS) system was used for the measurement of LA detection. The system was consisted by a system controller (CBM- 20A; Shimadzu Corporation), pump (LC- 20 AD; Shimadzu Corporation), auto-sampler (SIL-20 AC; Shimadzu Corporation), column oven (CTO-20 AC; Shimadzu Corporation), detector (3,200 QTRAP; AB Sciex, Tokyo, Japan), and analysis software (Analyst^®^ version 1.4.2; Shimadzu Corporation). The column and the guard column were Shodex^®^ ODP2 HP-2B 2.0 mm × 50 mm and ODP2 HPG-2A 2.0 mm × 10 mm, respectively (Showa Denko, Tokyo, Japan). The column temperature was set to 40°C, and the flow rat of mobile phase [isocratic mode, a mixture of 0.1% formic acid in water and acetonitrile (7:3)] was set to 0.2 mL/min. LA ionization was conducted by electrospray ionization, and the measured molecular weight of LA was set to m/z 605.30 for the precursor ion and m/z 249.00 for the production. The ion spray voltage was 5,000 V, the nebulizer gas pressure was 80 psi, the drying gas flow rate was 10 L/min, and the drying gas temperature was 600°C. The lower limit of quantification of this assay was 1.0 ng/mL. As an internal standard of betamethasone valerate was used for the LA assay. A standard curve was prepared with spiked plasma for the detection of blood concentration and without spiked plasma for the detection of LA release with standard LA over the range of 1.0 to 1.0 × 10^5^ ng/mL.

### 2.10 Statistical analysis

JMP^®^ Pro software (ver. 16.0.0, SAS Institute Inc., Cary, NC, United States) was used for statistical analyses. One-way ANOVA and Tukey’s honestly significant difference *post hoc* analysis was used to determine statistical significance (*p* < 0.05). More than three replicates, except for SAXS analysis, were conducted and all data were expressed as mean with standard deviation.

## 3 Results

### 3.1 Polarized microscope observation results of prepared formulations

**FIGURE 1:**
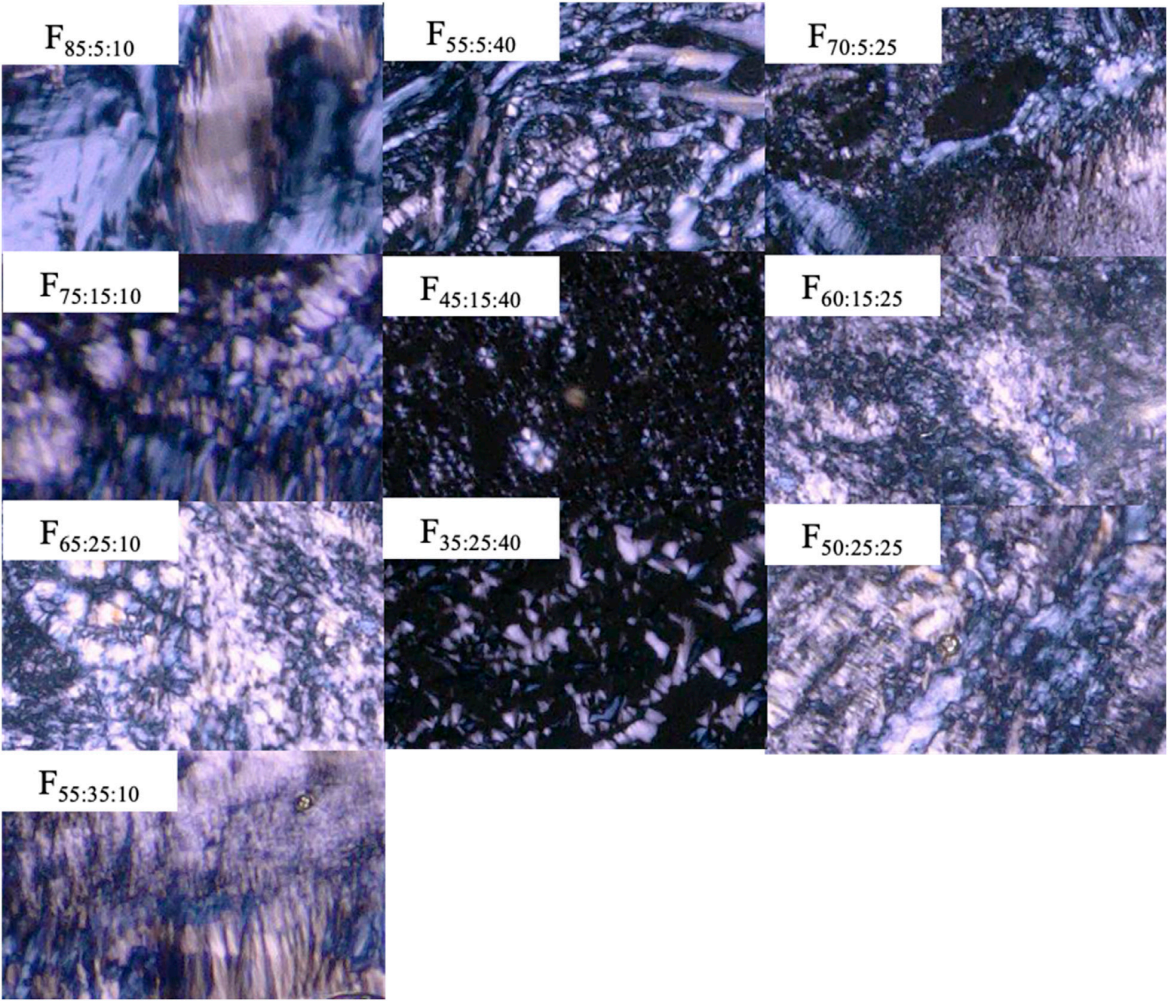
Polarized images of prepared formulations after mixing with water.

Polarized microscope observation was used for formulations consisting of SPC/DOPG/Span 85 at different proportions. Non-polarized images were observed for all formulations before contacting water (data not shown). Non-polarized images were obtained for formulations containing more than 50% Span 85, even after contacting water (F_25:25:50_ and F_12.5:25:62.5_). In addition, higher viscosity, which made it difficult to pass through a 23 G needle, was confirmed with increasing DOPG content (>50%) in the formulation (F_25:50:25_ and F_12.5:75:12.5_). Figure 1 shows a polarized image of the prepared formulations after swelling with water. All formulations for which polarization images obtained were injectable.

### 3.2 SAXS analysis

**FIGURE 2:**
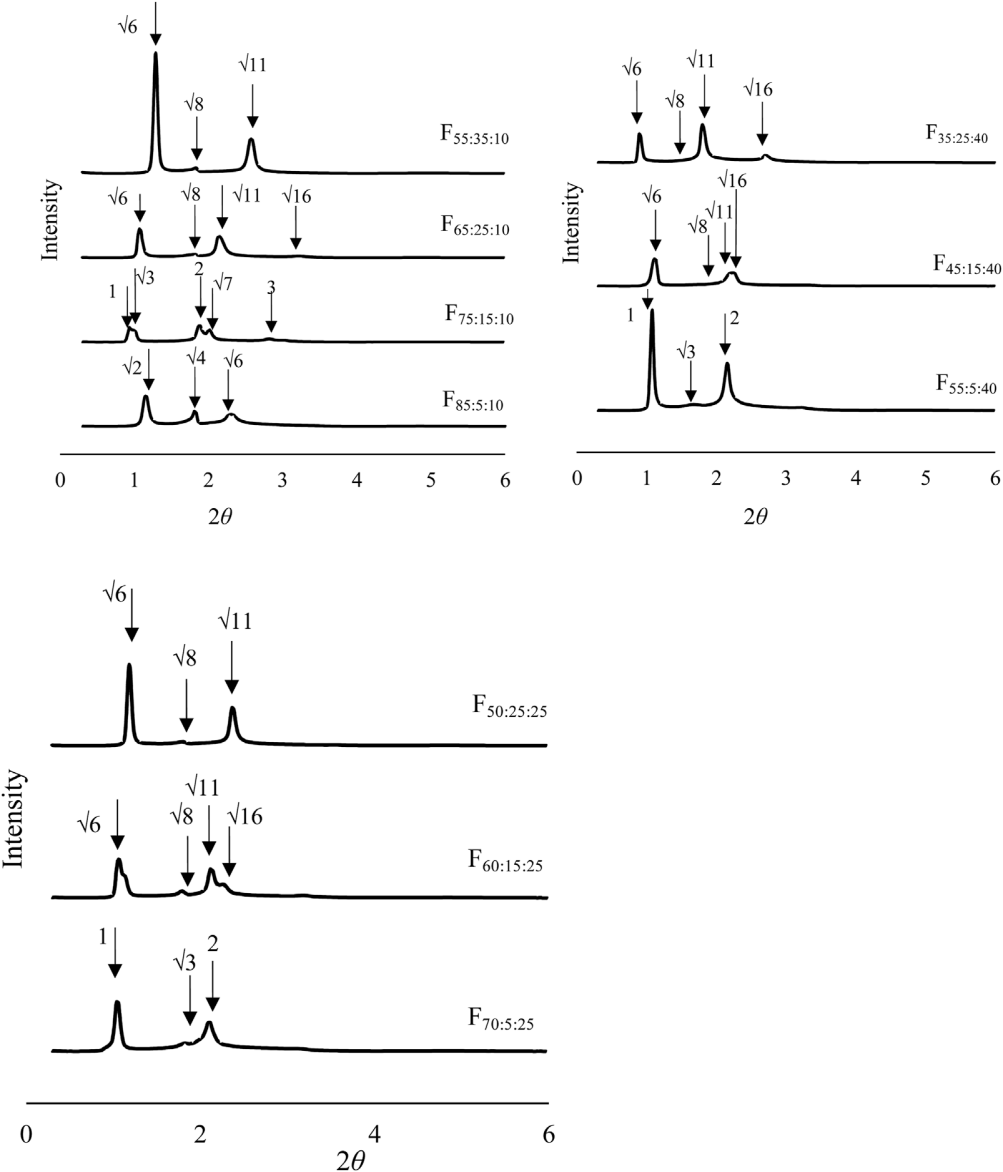
Structural analysis of the formulations after contacting water using small angle X-ray scattering analysis.

SAXS analysis was investigated for formulations that showed polarization images. Figure 2 shows the SAXS analysis results of the prepared formulations after contacting water. SAXS peaks were confirmed for all formulations. The spacing ratio of F_85:5:10_ was √2:2:√6:√8, whereas F_70:5:25_, F_55:5:40_, and F_75:15:10_ were 1:√3:2:√7, and the other formulations had 1:√3:2:√7. According to the diffraction patterns, the observed formulations had primitive cubic (*Im3m*), inverted hexagonal (*H2*), and reversed discontinuous micellar cubic (*Fd3m*) structures*,* respectively. The constructed NLLC structure changed from *Im3m* to *H2* or *Fd3m* according to increases in DOPG or Span 85 contents in the formulations. Interplanar spacing calculated from the Bragg equation is shown in Table 2. A larger interplanar spacing was confirmed with F_55:5:40_, which had *H2* structure, and the interplanar spacing was also changed by increasing Span 85 content in the formulation among the same structures.

**TABLE 2:** Observed structures of formulations after swelling with water and interplanar spacing (*d*) calculated using the Bragg equation.

Formulation code	Structure	*d* (nm)
F_85:5:10_	*Im3m*	2.75
F_70:5:25_	*H2*	4.12
F_55:5:40_	*H2*	4.63
F_75:15:10_	*H2*	3.02
F_60:15:25_	*Fd3m*	3.51
F_45:15:40_	*Fd3m*	3.87
F_65:25:10_	*Fd3m*	2.61
F_50:25:25_	*Fd3m*	3.23
F_35:25:40_	*Fd3m*	2.78
F_55:35:10_	*Fd3m*	1.29

The italic value represents primitive cubic (Im3m), inverted hexagonal (H2), and reversed discontinuous micellar cubic (Fd3m) structures.

### 3.3 *In vitro* release experiment

**FIGURE 3:**
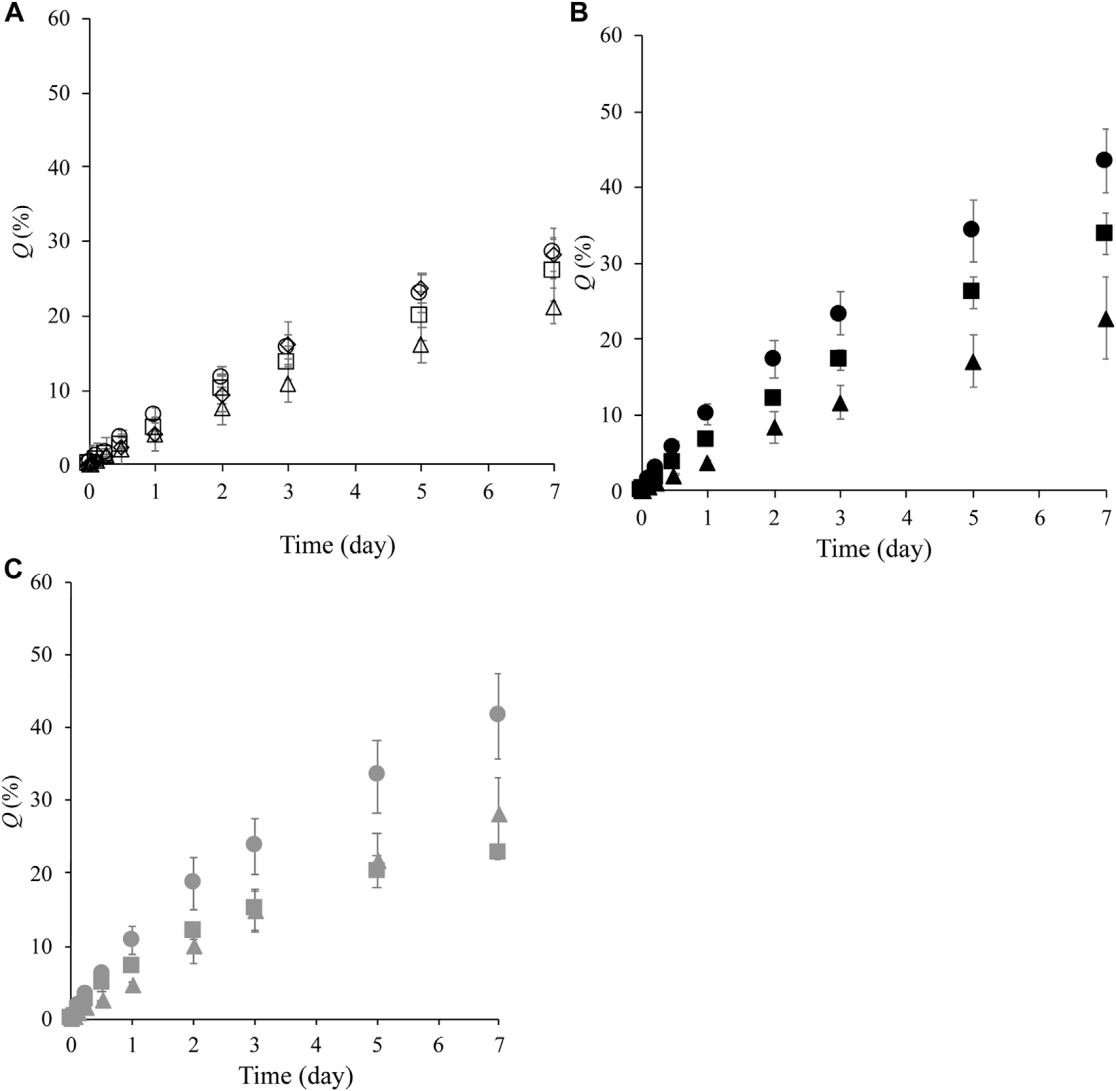
LA release profile from formulations over 7 days. **(A)**: release profile from F_85:5:10_ (○), F_75:15:10_ ( ), F_65:25:10_ (△), and F_55:35:10_ (◇), **(B)**: release profile from F_55:5:40_ (●), F_45:15:40_ (■), and F_35:25:40_ (▲), **(C)**: release profile from F_70:5:25_ (●), F_60:15:25_ (■), and F_50:25:25_ (▲). Each value shows the mean ± S.D. (*n* = 3).

Figure 3 shows the cumulative amount of LA released over 7 days, *Q*, from the prepared formulations. LA released from the formulations constructed of NLLC structures after contacting water was investigated. All formulations exhibited release behavior with more than 50% of LA remaining in the formulation after 7 days. Almost the same *Q*_7d_ value was observed in formulations containing 10% Span 85, F_85:5:10_, F_75:15:10_, F_65:25:10_, and F_55:35:10_, independent of increasing DOPG or decreasing SPC contents in the formulations (Figure 3A). On the other hand, LA release was decreased with an increase in DOPG content at 40% Span 85; 5% DOPG content formulation, F_55:5:40_, had *Q*_7d_ of 44%, 15% DOPG content formulation, F_45:15:40_, had *Q*_7d_ of 33%, and 25% DOPG content formulation, F_35:25:40_, had *Q*_7d_ of 23% (Figure 3B). With 25% Span 85 containing formulations, F_70:5:25_ showed a higher *Q*_7d_ value compared with other formulations F_60:15:25_ and F_50:25:25_, and these F_60:15:25_ and F_50:25:25_ formulations showed almost the same *Q*_7d_ value although they had different DOPG or decreasing SPC contents (Figure 3C).

### 3.4 *In vivo* experiment

**FIGURE 4:**
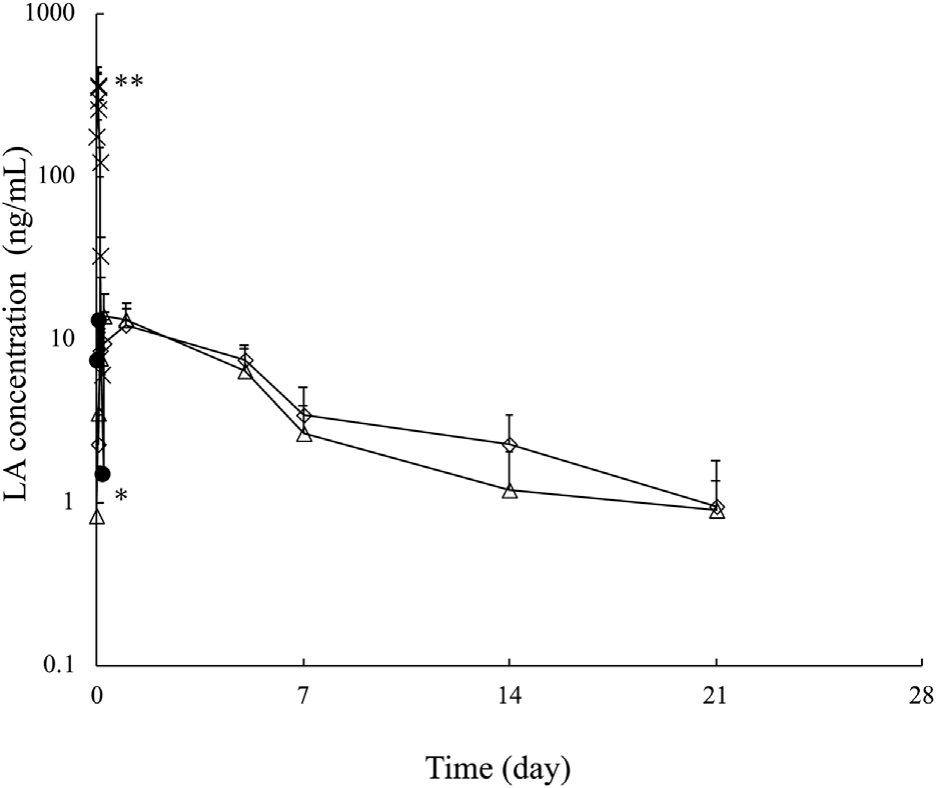
Plasma LA concentration–time profile over 21 days after subcutaneous injection of LA solution ( ), F_65:25:10_ (△), F_55:35:10_ (◇), and F_55:5:40_ (●). Each point shows the mean ± S.E. (*n* = 3–5). **p < 0.001 LA solution vs. the other formulations at 0.04 h, *p < 0.05 F_55:5:40_ vs. F_65:25:10_ and F_55:35:10_ at 1 day.

Figure 4 shows the blood concentration–time profile of LA after *s.c.* administration of the formulations F_65:25:10_, F_55:35:10_, F_55:5:40_, and LA solution. The formulations F_65:25:10_ and F_55:35:10_, which had the lowest and highest LA release levels, respectively, were selected for *in vivo* experiments. In addition, F_55:5:40_ was also selected to evaluate the effect of Span 85 content in the formation on the blood concentration–time profile was investigated compared with F_55:35:10,_ although almost the same *Q*_7d_ values between them were obtained in *in vitro* release experiments.

When LA solution was given by *s.c.* administration, a higher LA concentration was confirmed, although the concentration reached the lower limitation of detection (1 ng/mL) 8 h after administration. On the other hand, the tested formulations exhibited a maintained LA concentration compared with its solution. However, when F_55:5:40_ was given by *s.c.* administration, LA was detected until 24 h after administration for 21 days. The other formulations with F_65:25:10_ and F_55:35:10_ maintained LA concentrations. These formulations showed different release properties in *in vitro* release experiments; however, the same LA release profiles were confirmed after *s.c.* administration. Further LA detection 21 days after *s.c.* administration was not performed due to the lower limit of quantification of LA in blood. The calculated *AUC*_21days_ was 0.61 ± 0.08 μg・h/mL for LA solution, 0.84 ± 0.12 μg•h/mL for F_55:5:40_, 1.47 ± 0.44 μg•h/mL for F_65:25:10_, and 2.2 ± 0.42 μg•h/mL for F_55:35:10_. Increased *AUC*_21days_ was confirmed by F_55:5:40_, F_65:25:10_, and F_55:35:10_ compared with LA solution. However, the improved *AUC*_21days_ effect was slight for F_55:5:40_ compared to F_65:25:10_ and F_55:35:10_.

### 3.5 Injectability force of the prepared formulation

**FIGURE 5:**
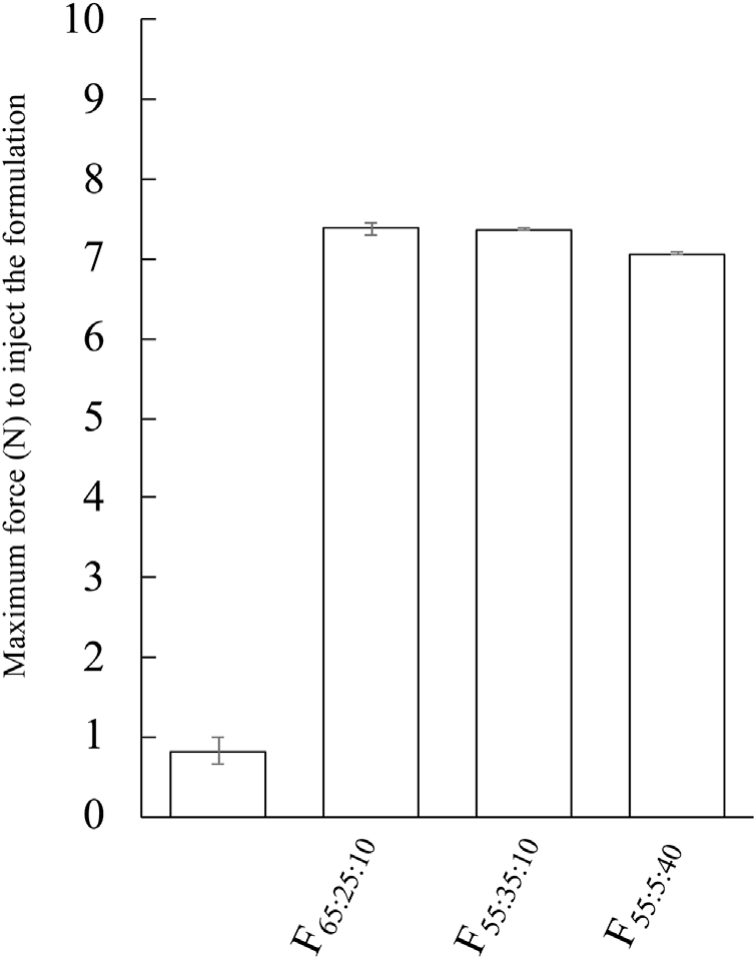
Evaluation of injectability force of F_65:25:10_, F_55:35:10_, and F_55:5:40_. The injectability force of the formulation was performed with 23G. Each value shows the mean ± S.D. (*n* = 3).

Figure 5 shows the injectability force of F_65:25:10_, F_55:35:10_, and F_55:5:40_. Water was selected as a control. The average force of the prepared formulation was higher than control, and their average forces were 7.38 N, 7.37 N, and 7.07 N for F_65:25:10_, F_55:35:10_, and F_55:5:40_, respectively. A slightly decreased maximum force was observed in F_55:5:40_ compared with the other prepared formulations.

## 4 Discussion

Injectable depot formulations are of increasing interest because they offer a reduction in the number of required injections and maintain therapeutic efficacy by stabilizing blood concentrations. These advantages help in reducing unwanted side effects. In addition, depot formulations have been used for many different medical controls (antipsychotic, substance abuse, and hormonal therapy drugs) (Chaudhary et al., 2019). Polymers and lipids are the major excipients used in depot formulations. PLGA, a commonly used polymer in depot formulations, has different molecular weights and copolymer compositions, providing a wide range of drug release control. However, PLGA formulations are difficult due to their complex manufacturing process, and lipid-based formulations have gained attention instead of PLGA formulation for delivering drugs for long-term-period, in addition to PLGA formulation. Oil-based (Wilkinson et al., 2022) and nanodispersion-based (Altamura et al., 2003) formulations, such as liposomes and emulsions, have been used as lipid-based depot formulations (Lim et al., 2022). Recently, *in situ* lipid-based crystal-forming system have been assessed (Mei et al., 2018; Li et al., 2019; Pineda-Hernandez et al., 2020). NLLC structures can be formed spontaneously by contacting aqueous fluid, and it may be possible to control the drug release of entrapped drugs from the constructed structure. Drug release controlled by a constructed non-lamellar structure and its interplanar distance have been reported (Clogston and Caffrey, 2005). In addition, entrapped drug stability in the constructed non-lamellar structure has also been reported (Boge et al., 2016). However, the normal viscosity of the formulations is increased by forming an NLLC. When microneedle systems and non-needle-free injection systems as well as conventional needles were used as administration devices into the skin, formulations with a lower viscosity are preferable. Thus, in the present study, *in situ* crystal-forming systems with a simple composition were investigated with amphiphilic components to provide sustained release.

Many researchers have reported drug release from NLLC nanoparticles, and NLLCs can slowly release entrapped drugs. Drugs can diffuse in the lamella phase along a planner bilayer, whereas in a cubic phase, they can diffuse three-dimensionally. On the other hand, a hexagonal phase has one-dimensional water parallel with the water cylinder, and hydrophilic drugs exist in the polar core of the inversed micellar cube. Therefore, in general, the release rate of drugs exhibit the following order; diffusion in the lamellar structure (*D*_lamellar_) > *D*_cubic phase_ > *D*_hexagonal phase_ > *D*_micellar cubic_ (Muszynski et al., 2018). In the present study, higher *Q*_7d_ values seen with F_70:5:25_ and F_55:5:40_, which exhibited *H*_*2*_ structures, were confirmed compared with formulations with an *Fd3m* structure (F_60:15:25_, F_45:15:40_, F_65:25:10_, F_50:25:25_, F_35:25:40_, and F_55:35:10_). Although this result corresponded with other reports, almost the same *Q*_7d_ value was confirmed in even *H*_*2*_ constructing formulations such as F_75:15:10_ compared with *Fd3m* constructing formulations. Electrostatic interactions between the charged drug and the polar group in the lipid component may also achieve drug release control (Lynch et al., 2003; Negrini et al., 2014). Therefore, in the present study, formulations containing higher DOPG were listed (Table 1), but no experiments were performed with those with more than 50% DOPG because of their high viscosity.

The F_75:15:10_ formulation had a higher proportion of DOPG, which has a positive charge at pH 7.4, compared with F_70:5:25_ and F_55:5:40_. Thus, negatively charged LA was thought to have a slower release rate. In addition, the F_85:5:10_ formulation showed a relatively lower release rate compared with the other formulations. The release rate depended on the constructed structure in addition to the size of the aqueous channel (Muszynski et al., 2018; Zhai et al., 2022) because, in the case of hydrophilic drugs, they must diffuse through an aqueous channel and cross the lipid membrane. Therefore, a smaller aqueous channel in F_85:5:10_ may be the reason for the slower LA release rate.

**FIGURE 6:**
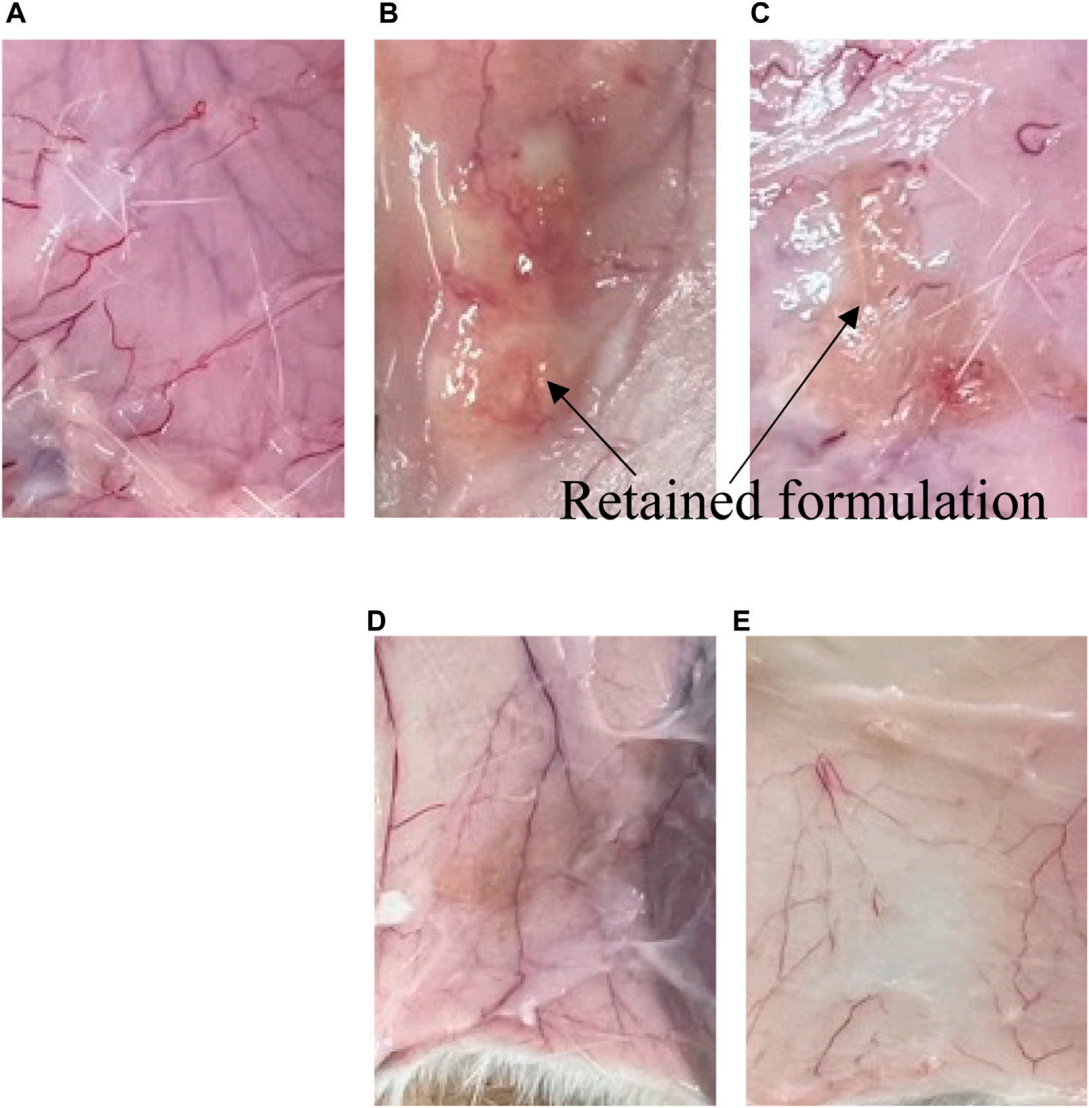
Remaining formulation at the injection site at 7 and 28 days after *s.c.* administration. **(A–C)** show 7 days after administration of **(A)** F_55:5:40_, **(B)** F_55:35:10_, and **(C)** F_65:25:10_, respectively. **(D,E)** show 28 days after administration of F_55:35:10_ and F_65:25:10_, respectively.

Generally, when the rate-limiting step for drug absorption with aqueous solubility is the rate of drug release; the release rate can be considered to be related to the blood concentration–time profile. However, no blood concentration differences were confirmed between F_55:35:10_ and F_65:25:10_, although these have different release profiles of LA. On the other hand, the blood concentration of LA after *s.c.* administration of F_55:5:40_, which had a faster LA release rate, displayed the lower limit of detection within 8 h. Figure 5 shows the observation results of the remaining formulation in the body 21 days after administration. The observation was performed by surgically incising around the administration site. Phospholipids and Span 85 were degraded by lipases and non-enzymatic hydrolytic processes (He et al., 2023). Biodegradation of the formulation causes erosion, and subsequent formulation reduction until sufficient excretion is reached. Therefore, the biodegradation process may also affect the drug release rate from the formulation, especially in formulations with sustained drug release. In the present study, retention of the formulation was confirmed 7 days and 28 days after administration. When the F_55:5:40_ formulation was administered, no retained formulation was observed (Figure 6A). On the other hand, the F_55:35:10_ and F_65:25:10_ formulations were observed 7 days after administration (Figures 6B, C, respectively), but these formulations were not clearly observed 28 days after administration (Figures 6D, E, respectively). Since blood concentration of LA was not detected 21 days after administration, only formulation excipients would remain at the injection site. No signs of irritation, inflammation, or any other apparent toxicity were observed grossly at the injection site. In addition, body weight loss was not observed in the present study 28 days after administration compared with just before administration.

Controlled drug release may be attributed to the time required for water penetration into the core of the administrated formulation. In *in vitro* release conditions, drug release can be achieved without enzymatic degradation. In addition, in *in vivo* conditions, the transition time to construct an NLLC structure might be delayed in the body because of limited interstitial fluid volume. When the F_55:5:40_ formulation was administered, the absence of retention was confirmed 7 days after administration. On the other hand, the F_55:35:10_ and F_65:25:10_ formulations, which showed almost the same *Q*_7d_ value, had a similar blood concentration–time profile. Therefore, it might be possible that the spread of components of the NLLC formulation at the administration site, which affects the construction of the NLLC structure, may be involved as a factor. This might be related to the lack of significant differences in blood concentration–time profiles between the F_55:35:10_ and F_65:25:10_ formulations.

When F_55:35:10_ and F_65:25:10_ formulations were administered, *AUC*_21days_ values were 2.2 ± 0.42 μg•h/mL and 1.47 ± 0.44 μg•h/mL, respectively. Compared with the *AUC*_21days_ after s.c. administration of LA solution, increased *AUC*_21days_ was confirmed in both formulations. Ki et al. (2014) have reported *AUC* value after *s.c.* administration of a commercial product of PLGA formulation. The calculated AUC was 3.7 μg•h/mL when the dose of 12.5 mg/kg was administered to rats (approximately 300 g body weight). As these *AUC* values were corrected for administrated LA dosage, the commercial product of PLGA formulation showed about 3 times higher *AUC*/dose value compared with that of F_55:35:10_. In the present study, Span 85 mixed with phospholipids was used to provide sustained release of LA by constructing NLLC formulations. Furthermore, the average maximum force requirement of these formulations was less than 10 N when 29G needle was used. Patki et al. (2021) reported on self-injection formulations with sustained release and concluded that formulations with an average maximum force of 10 N or less are very easy to inject. F_55:35:10_ and F_65:25:10_ showed that around 7 N when the formulation was injected with 29G needle. In our previous report with NLLC forming lipid of MGE, which has CPP>1 (Okada et al., 2021), a maintained the blood concentration of LA over 21 days or more after administration. However, there was a problem for injectability because they needed a higher expulsion force (higher than 10 N, when the formulation was tested according to the experiment method in 2.7). On the other hand, in the present study, phospholipid formulation showed a maintained concentration of LA until 21 days with a good injectability. Molecular interaction is generally related to viscosity (Pichot et al., 2013), so the difference in injectability properties of the prepared formulation would be related to additives and components in the formulation.

Examination of tail chain length on the effect of the constructed NLLC structure revealed that an increase in CPP value induced phase transition from lamellar or cubic to reverse hexagonal structures (van ’t Hag et al., 2017; Ruela et al., 2016). Because Span 85 has a large lipophilic moiety similar to medium- and long-chain triacylglycerol, thus phase transition to increasing negative interfacial curvature may occur by adding it to a formulation. However, *AUC*/dose value in our prepared formulation was lower than that in the commercial product of PLGA formulation, suggesting that further formulation development to improve bioavailability should be necessary.

## 5 Conclusion

In the present study, *in situ* NLLC constructed formulations by contacting water were developed using a ternary mixture of SPC, DOPG, and Span 85. In particular, the negative curvature was increased with an increase in the amount of Span 85 in the formulation, and an *Fd3m* structure was obtained with a sustained release of LA. A maintained blood concentration of LA over 21 day was confirmed by *s.c.* administration of the formulation. However, the elimination of components of the formulation by enzymatic degradation might need to be considered together with *in vitro* release experiments for the evaluation of depot formulations with a sustained release. In the present study, evaluation of systemic and local toxicity after *s.c.* administration was not performed.

This lipid-based formulation can be prepared as a depot formulation with sustained release of the encapsulated drug more simply than polymer-based formulations. Further investigations, such as the detailed mechanism of self-assembly of the NLLC structure by analyzing lipid-lipid and lipid-drug interactions, as well as rheological studies to enable injection, will be useful in the selection of lipid compositions capable of forming the NLLC structures *in situ*. Although bioavailability equivalent to commercial PLGA products would be required to indicate the usefulness of the phospholipid and Span 85-based formulation, these investigations will be helpful for developing injectable depot formulations with controllable release of drugs such as peptides and proteins.

## Data Availability

The raw data supporting the conclusion of this article will be made available by the authors, without undue reservation.
